# Optimization of Docetaxel Loading Conditions in Liposomes: proposing potential products for metastatic breast carcinoma chemotherapy

**DOI:** 10.1038/s41598-020-62501-1

**Published:** 2020-03-27

**Authors:** Roghayyeh Vakili-Ghartavol, Seyed Mahdi Rezayat, Reza Faridi-Majidi, Kayvan Sadri, Mahmoud Reza Jaafari

**Affiliations:** 1 Department of Medical Nanotechnology, School of Advanced Technologies in Medicine, Tehran University of Medical Sciences, Tehran, Iran; 2 Department of Pharmacology, School of Medicine, Tehran University of Medical Sciences, Tehran, Iran; 3 Nuclear Medicine Research Center, Mashhad University of Medical Sciences, Mashhad, 98451-3546 Iran; 4 Nanotechnology Research Center, Pharmaceutical Technology Institute, Mashhad University of Medical Sciences, Mashhad, Iran; 5 Department of Pharmaceutical Nanotechnology, School of Pharmacy, Mashhad University of Medical Sciences, Mashhad, Iran

**Keywords:** Chemotherapy, Drug delivery, Drug development, Breast cancer

## Abstract

Docetaxel (DTX) was loaded in nanoliposomes based on a new remote loading method using mannitol and acetic acid as hydration buffer. DTX loading conditions were optimized, and the final formulations were prepared according to the best parameters which were HSPC/mPEG2000-DSPE/Chol (F1), HSPC/mPEG2000-DSPE/DPPG/Chol (F2), HSPC/mPEG2000-DSPE/DSPG/Chol (F3), at molar ratios of 85/5/10, 80/5/5/10, 80/5/5/10, respectively. DTX-liposomes were found of desired size (~115 nm) and homogeneity (PDI ≤ 0.2), high drug encapsulation efficacy (34–67%) and DTX concentration, and favorable stability. Passive loaded counterparts liposomes showed three times lower encapsulation efficacy compared to the remote loaded liposomes. The drug release of remote loaded liposomes in plasma 50% was significantly more controlled and less in comparison with their passive loaded counterparts (p < 0.0001). The IC50 values of formulations were determined on MCF-7, 4T1, TUBO, NIH/3T3 cell lines. The biodistribution of iodinated docetaxel as free or liposomal form exhibited significantly greater accumulation of DTX-liposomes in tumors than that of free docetaxel due to the EPR effect. *In vivo* experiment with BALB/c mice bearing 4T1 or TUBO breast carcinoma tumors also showed that DTX-liposomes could significantly delay tumor growth and prolonged the survival time in comparison with control and Taxotere groups at the similar dose of 8 mg/kg. F1 and F2 formulations were stable and showed good anti-tumor activity and merit further investigation.

## Introduction

Taxanes have extremely low solubility in water and pharmaceutical concerns caused by their bulky polycyclic structure^1,2^. Nanocarriers, including liposomes, micelles, and polymeric or inorganic nanoparticles have been developed to prepare a higher therapeutic efficacy, lower toxicity, and controlled delivery of chemotherapeutic agents^1,3–8^. Among them, liposomes, composed of phospholipids, have been explored for their use in clinically approved formulations for more than five decades^6,9–15^. Liposomes, compared with other nanocarriers, present specific advantages and promising capabilities in delivering a plethora of otherwise inefficient drugs by modifying their physicochemical characteristics and biodistribution, and by reducing the toxic effects of drugs^6,16,17^. Moreover, chemical and biological stability under different storage conditions of agents and during blood circulation, and biocompatible characteristics recommend them as carriers for therapeutic agents^6,9,17,18^. There are currently two liposomal formulations of docetaxel (DTX) in clinical development. LE-DT (NeoPharm, Inc.) and ATI-1123 (Azaya Therapeutics, Inc.) have undergone phase I studies and appears to be well tolerated (ClinicalTrials.gov in May 2019).

In general, drug loading into the liposomes is attained by either passive or active (remote) methods^19^. In passive loading method, dried thin film is hydrated in aqueous solutions containing the drug of interest. In contrast, remote loading technique is loading of a drug into performed liposome and initiated by co-mixing a liposome suspension with a solution of drug and drug internalization is typically driven by a transmembrane electrochemical gradient^7,19–21^. This method can result in high drug encapsulation efficiency, minimal wastage of precious chemotherapeutic agents (< 5%) in manufacturing, and enhanced stability in during storage and patient administration^19,22^. In this work, we described a novel method for remote liposomal loading of docetaxel as a hydrophobic drug and then delivering them to tumor via passive targeting and the enhanced permeability and retention effect (EPR).

Accumulating body of studies on the transport of macromolecules into tumor tissues have demonstrated that blood plasma components, including proteins, macromolecules, nanoparticles, lipidic particles, and other soluble particles could extravasate through leaky tumor vasculature to enter within the interstitial space of tumors and accumulate there due to the inability of lymphatic drainage in tumor tissues. This phenomenon was later termed the EPR effect and paved the way for the passive targeting of tumors using nano sized particles^23–27^. We aimed at developing liposomal docetaxel based on remote loading process and showing sustained and controlled drug delivery with decreased toxicity and improved efficacy. Here, we evaluated if our liposomal DTX could achieve higher therapeutic efficacy against 4T1 and TUBO cells compare to Taxotere. Using the mice bearing 4T1 or TUBO breast cancer cell lines, which were used in Taxotere development, we assessed the Median survival time (MST), Time to reach end point (TTE), and Tumor growth delay (TGD), Increase life span (ILS), and therapeutic efficacies of liposomal DTX compared to Taxotere and PBS. Biodistribution of NLs was determined using iodinated DTX.

## Results and Discussions

### Physicochemical characterization of DTX loaded nanoliposomes

In this study, different methods based on passive and remote loading were performed to encapsulate DTX in the liposomes. Among different methods, remote loading based on mannitol and acetic acid provided optimal encapsulation efficacy. The formulations of HSPC/mPEG2000-DSPE/Chol (F1), HSPC/mPEG2000-DSPE/DPPG/Chol (F2) and HSPC/mPEG2000-DSPE/DSPG/Chol (F3) at molar ratios of 85/5/10, 80/5/5/10, 80/5/5/10, respectively (total lipid: 100 mM) exhibited the highest entrapment ability with a desired stability. Therefore, they were selected for *in vitro* and *in vivo* studies (Table 1).

**Table 1:** Size, ζ potential, DTX content and drug encapsulation efficiency of DTX loaded NLs.

Formulation (Molar Ratio)	Z-Average (nm)	PDI^a^	ζ -potential (mV)	mg of DTX/ml	EE^b^ (%)
**Active loaded liposomes**					
F1_a_: HSPC/mPEG2000-DSPE/Chol (85/5/10)	109.8 ± 2.8	0.16 ± 0.00	−10.8 ± 0.2	1.7 ± 0.2	53.12 ± 8.8
F2_a_: HSPC/mPEG2000-DSPE/DPPG/Chol (85/5/5/10)	115.1 ± 2.8	0.17 ± 0.03	−12.4 ± 0.4	1.1 ± 0.1	34.3 ± 4.4
F3_a_: HSPC/mPEG2000-DSPE/DSPG/Chol (85/5/5/10)	116 ± 1.9	0.11 ± 0.00	−13.8 ± 0.0	2.15 ± 0.2	67.18 ± 6.6
**Passive loaded liposomes**					
F1_p_: HSPC/mPEG2000-DSPE/Chol (85/5/10)	125.0	0.087	−10.3	0.64 ± 0.2	20 ± 6.2
F2_p_: HSPC/mPEG2000-DSPE/DPPG/Chol (85/5/5/10)	139.0	0.156	−10.9	0.57 ± 0.15	18 ± 4.7
F3_p_: HSPC/mPEG2000-DSPE/DSPG/Chol (85/5/5/10)	108.4	0.178	−13.7	0.83 ± 0.3	26 ± 0.94

Each value depicts mean ± standard deviation (n = 3).

^a^Polydispersity index, ^b^Encapsulation Efficacy.

Subscripts are abbreviations of active and passive loaded methods, respectively.

### Size, zeta potential, DTX content, and drug encapsulation efficiency

For different formulations, we measured the mean liposome diameter, size distribution, and zeta potential to ensure that all three NL formulations had the acceptable properties for delivery to tumor based on EPR effect. The sizes of liposomes distributed from 109 nm to 120 nm with a polydispersity index <0.2, implying uniform distribution of all groups of liposomes (Table 1). The particle size is a key parameter determining nanoparticle distribution and therefore bioavailability^28^. Experiments using liposomes of different mean size suggest that vesicles in the size range of 20–200 nm can more effectively extravasate and accumulate inside the tumor tissue and inflammatory sites^29–31^. Therefore, the size of the DTX loaded NLs prepared in this research was in the size range favoring the therapeutic benefits for tumor treating. The zeta potential of F1, F2, and F3 formulations were −10.8 mV, −12.1 mV, −13.8 mV, respectively. Zeta potential is important for the particle stability, and influences the *in vivo* fate of the liposomes^32–34^. It can be used to define whether a charged active material is entrapped within the center of the nanoparticle or on the surface^35^. The F2 and F3 formulations became more negative charged (approximately 2–3 mV) compared to the F1 formulation which implied that the negative charges of dipalmitoyl-sn-glycero and distearoyl-sn-glycero domains were partly presented by the DPPG and DSPG residues, respectively. Regarding this issue, DTX could have no electrostatic interaction with the surface of negatively charged liposomes due to its partial negative charges (isoelectric pHs: 4.6, 6.5, and 7.4) in hydration buffers at pH 7.00 (https://chemaxon.com). Since, some studies showed that in the conventionally loaded processes, the incorporation of the DTX is only 0.3 to 0.7 mg/mL^36^, it was necessary to determine DTX positioning in the formulations. To examine the inner aqueous phase of liposomes, we examined maximum solubility of DTX in solutions of hydration buffer, dialysis buffer, and distilled water. For this, we prepared saturated solutions of docetaxel using these buffer solutions. After filtering through 0.2 µm microbial filters, the filtrates were assayed for DTX concentration. Our results demonstrated that DTX is similarly dissolved in all three solutions (Supplementary Table S1). Therefore, it could be presumable that some rafts formation in remote loaded liposomes is responsible for good drug incorporation and solution of mannitol and acetic acid might change the fluidity of the bilayer (internal leaflet) enabling more stable drug incorporation. Lipid rafts are small (10−200 nm), heterogeneous, highly dynamic, tightly packed liquid ordered state microdomains in bilayers that are generally enriched in both lipids with predominantly saturated acyl chains and sterol. They are thought to coexist with disordered membrane domains that are rich in lipids with unsaturated acyl chains^37–39^. Since the incubation temperature of 67 °C could achieve maximum drug loading, it is concluded that the extent of microdomain formation may be temperature-dependent. This finding was consistent with the results of the some other studies which demonstrated the effect of temperature on lipid rafts formation^40,41^.

The most important finding was the results of measurement for DTX content (1.7 ± 0.28, 1.1 ± 0.14, and 2.15 ± 0.21 mg/ml DTX entrapment) and encapsulation efficiency (53.12 ± 8.8%, 34.3 ± 4.4%, and 67.18 ± 6.6% EE) for F1, F2, and F3 nanoliposomes, respectively (Table 1). This high amount in drug loading could be a sign for successful remote loading and good encapsulation of DTX into the liposomes^42,43^.

These results are confirmed with the findings of some other studies, which showed remote drug loading into nanoliposomes is in most cases the best method for achieving high concentrations of active pharmaceutical ingredients per nanoliposome that enables therapeutically viable nanodrugs^44–46^.

**Figure 1:**
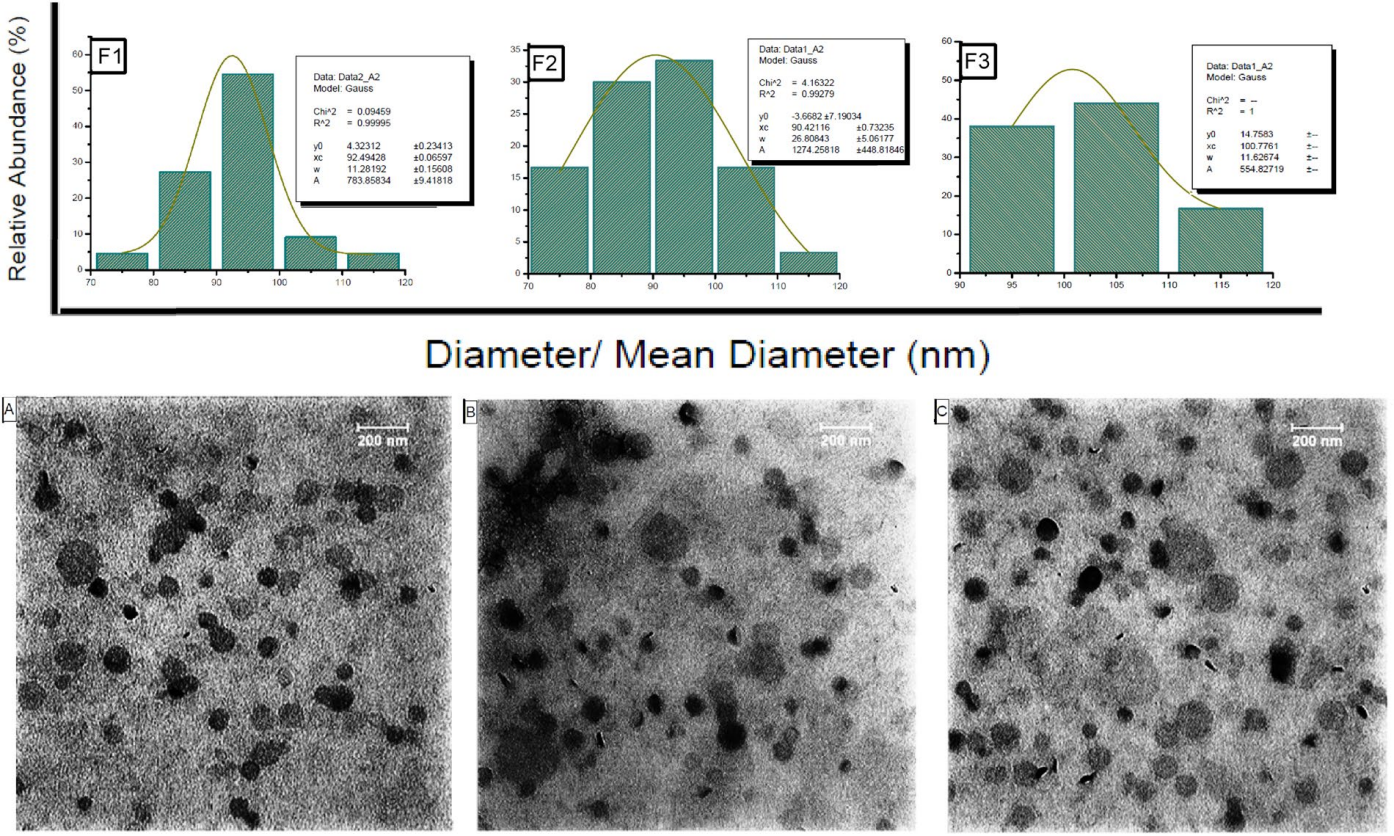
Size distribution, TEM images of DTX loaded F1, F2, and F3 nanoliposomes.

Surface morphology in all three types of drug loaded NLs was found by TEM to be spherical in shape. The particle size obtained from the DLS can also be confirmed from the TEM image (Fig. 1).

Accumulating body of studies aimed to encapsulate DTX inside the internal water compartment of the liposomes to retain the drug with only negligible leakage over a prolonged period of time *in vitro*^36,47,48^. Since docetaxel exhibits poor water-solubility, most of these studies papered hydrophilic DTX derivatives which could be actively loaded at high drug-to-lipid ratios into nanoliposomes. However, there is a concern about potential reductions in efficacy in the use of derivatives.

### *In vitro* drug release

**Figure 2:**
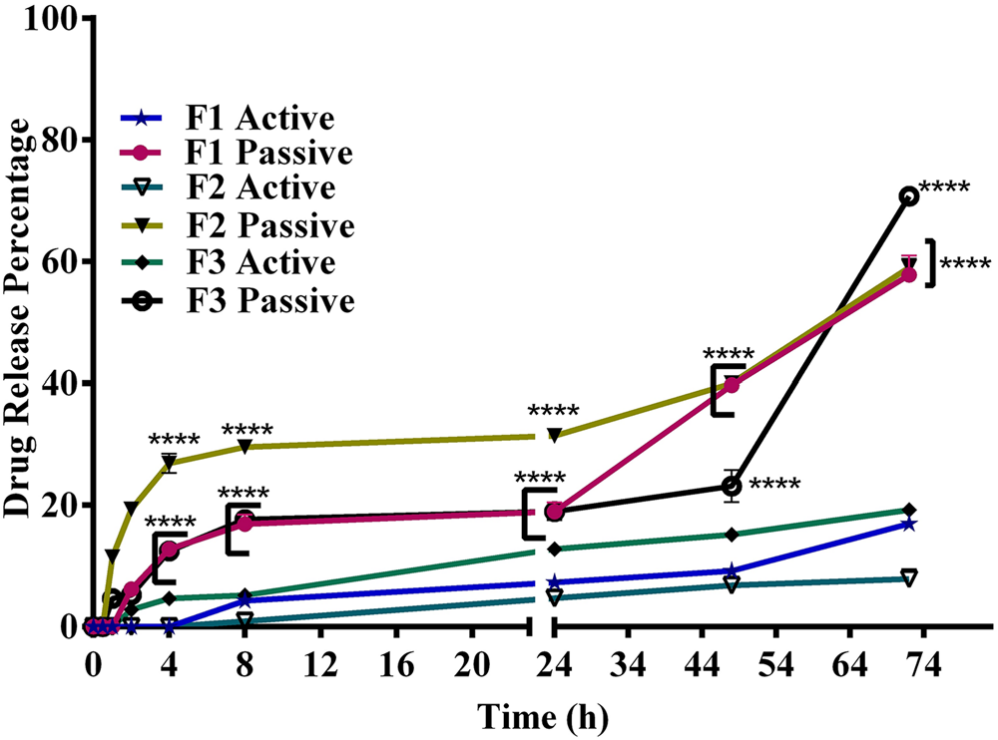
The DTX release profile of active loaded nanoliposomes in comparison with passive loaded counterparts in 50% fresh human plasma. Drug release from active loaded nanolipsomes was significantly lower than that of passive loaded nanoliposomes (p < 0.0001). The error bars were obtained from duplicate samples.

Drug release profile is usually evaluated to prove formulation quality, provide reference for dosage regimen, and predict the efficacy of the formulations *in vivo*^49^. Figure 2 shows the *in vitro* drug release profiles of remote loaded liposomes versus passive loaded liposomes in 50% fresh human plasma in 72 h which display no burst release for none of liposomes. However, remote loaded liposomes showed significantly more controlled release of docetaxel in comparison with their passive loaded counterparts at different time points (p < 0.0001). The release of DTX from formulations in plasma 50% was very low for remote loaded liposomes and it reached up to around 18% for F1 and F3 formulations, and 8% for F2 formulation, after 72 hours. However, for passive loaded liposomes the release reached up to around 58% for F1 and F2 formulations, and 70% for F3 formulation, after 72 hours.

**Figure 3:**
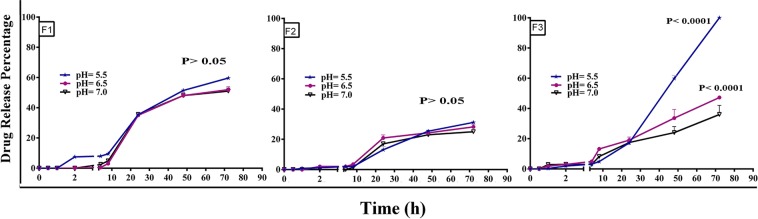
*In vitro* drug release profile of F1, F2 and F3 nanoliposomes at pHs of 5.5, 6.5, and 7.00. At different pHs, F1 and F2 represent a pH independent DTX release (p > 0.05). DTX release from F3 nanolipsomes at both pHs of 5.5 and 6.5 was significantly higher than that of pH 7.00 (p < 0.0001). The error bars were obtained from triplicate samples.

Moreover, drug release profiles were evaluated for remote loaded nanoliposomes at different pHs of 5.5, 6.5, as well as 7.00 (Fig. 3). The release of DTX from formulations was very low in the first 8 hours and it was less than 5% for all formulation at various pHs. There was a slightly difference in DTX release between formulations in the first 8 h. They are followed by a steady release rate; for F1 formulation the percent of release reached up to 55% after 72 hours at various pH, for F2 formulation the release reached up to around 30% at various pH, for F3 formulation the release was reached to around 42% at pH 7.0 and 6.5; however, there was an outstanding release for F3 NLs at pH of 5.5 and it was reached up to 100% after 72 hours. Moreover, compared with F1 and F2 nanoliposomes, drug release from F3 formulation is found pH dependent and the acidic condition could significantly speed the DTX release from F3 nanoliposomes (p < 0.0001). Nevertheless, F1 and F2 show a pH-independent DTX release during the time. These results demonstrate the composition of lipids in liposomes could affect the release behavior of the drug from liposomes as consistent with the findings of some other studies^50,51^.

The distinct behavior of F3 formulations can be attributed to the following reasons: (1) as the hydrocarbon chain length of lipid components is increased, Van der Waals interactions between the lipid chains and DTX become stronger requiring more energy to be disrupted. Therefore, DSPG in the F3 formulation compared with DPPG in the F2 formulation retains relatively considerable amounts of DTX with a strong association near or within the liposomal bilayer membrane. (2) The surface charge and hydration of DSPG in pH variation from 7 to 6.5 may slightly change. It is therefore reasonable the association between DSPG and encapsulated DTX in the bilayer can only be affected and then released. However, pH variation from 7.00 to 5.5 could result in remarkable changes in DSPG such that make the liposome membrane unstable, thereby promoting drug fast release from F3 nanoliposomes. Moreover, the results of release profile monitoring at pH 5.5 and 6.5 for F1 and F2 formulations are in line to their trends observed in pH 7.00 and do not statistically represent significant difference than that of physiological pH.

Moreover, it could be concluded that all of the remote loaded liposomes are stable in the plasma after i.v. injection as long as they are circulating in the blood (Fig. 2), and then gradually accumulate in the tumor environment by EPR. Among these three formulations, F2 nanoliposomes ideally show slower drug release thus it is predicted to effectively deliver DTX to tumor site.

### Stability tests

We followed the z average (ZA), PDI, ζ potential (ζ-P), and leakage stability of nanoliposomes immediately after their preparation and after storage for 1, 3, 6 months at 4 °C. As shown in Table 2, all three types of liposomes indicated no substantial changes in hydrodynamic size, PDI, and ζ potential within 6 months, indicating that the nanoliposomes have a very high stability. However, leakage analyses of NLs did not show similar patterns. In general, all formulations displayed an acceptable stability at 4 °C after 1 month. The best stability was achieved from F1 NLs which maintained 71.76% and 54.6% of the initial amount of the encapsulated drug, after 3 and 6 months, respectively. These results suggest that F1 NLs has a good stability within 6 month. Moreover, F2 and F3 NLs released about 70% encapsulated drug after 3 and 6 months which showed their lower leakage stability in comparison with F1 formulation.

**Table 2:** Results of stability test for docetaxel loaded NLs.

Time	0 Month				1 Month				3 Months				6 Months			
	ZA^a^	PDI^b^	ζ -P	DTX (mg/ml)	ZA	PDI	ζ –P^c^	DTX (mg/ml)	ZA	PDI	ζ -P	DTX (mg/ml)	ZA	PDI	ζ -P	DTX (mg/ml)
F_1_^d^	109.6	0.165	−10.8	1.6	112.8	0.164	−10.6	1.6	107.2	0.178	−11	1.23	106	0.16	−9.26	1.01
F_2_	118.1	0.198	−13.8	1.2	114.3	0.172	−13.9	1	115.6	0.168	−13.9	0.43	115.2	0.172	−10.1	0.32
F_3_	113.1	0.145	−12.1	2.3	117.1	0.21	−12.8	1.59	109.5	0.168	−9.85	0.61	108.9	0.18	−8.07	0.42

^a^Z average, ^b^Polydispersity index, ^c^ζ –potential, ^d^Formulation.

### *In vitro* cytotoxicity of NLs

Table 3 shows IC_50_ of DTX formulated in Taxotere and nanoliposomes on NIH/3T3, 4T1, TUBO, and MCF-7 cells after 24, 48 and 72 hours cell culture at the same DTX dose. Three conclusions could be made from this table: (1) when cell lines were treated with the nanoliposomes, mortality effect was comparable to that of cells treated with Taxotere formulation after 72 hour cell incubation (p > 0.05). However, in the liposomes, DTX has sustained and controlled release profile and it can be therefore concluded that nanoliposomal formulations would have even better effects than the clinical formulation; Taxotere in a long time periods. (2) Each of these treatments has a different effect on various cells at different time periods which may be contributed to the controlled drug release manner of the nanoliposome formulations, cellular internalization rate of DTX and/or formulations in to the cells, and resistance to the DTX due to causes such as up-regulation of protein transporter pumps by cancer cells. (3) Cytotoxicity values of NIH/3T3, 4T1, and TUBO cell lines after 24 h incubation with the F3 formulation were significantly more effective than the Taxotere formulation. This finding is in agreement with release study and fast releasing of DTX from F3 formulation.

**Table 3:** IC_50_ of DTX formulated in Taxotere and nanoliposomes on NIH/3T3, 4T1, TUBO, and MCF-7 cells after 24, 48 and 72 hour cell culture at the same DTX dose (n = 6, mean ± SD).

Cell Lines	Incubation Time (hour)	IC50 (ug/ml)			
NIH	24	**Taxotere**	**F1**	**F2**	**F3**
		27.01	128.83	77.96	26.13
	48	27.73	22.77	32.95	11.5
	72	26.7	13.42	12.15	5.7
4T1	24	53.27	304.96	154.5	67.44
	48	12.39	40.15	27.27	20.9
	72	13	8.7	14.13	14
TUBO	24	17.5	223.5	153	9.5
	48	14.32	39.63	17.8	0.84
	72	4.7	4.1	0.5	0.0312
MCF-7	24	48.35	167.72	170.88	22.99
	48	24	10.75	24.98	18.11
	72	14.35	1.9	15.9	6.7

### Animal studies

#### Chemotherapy study

**Figure 4:**
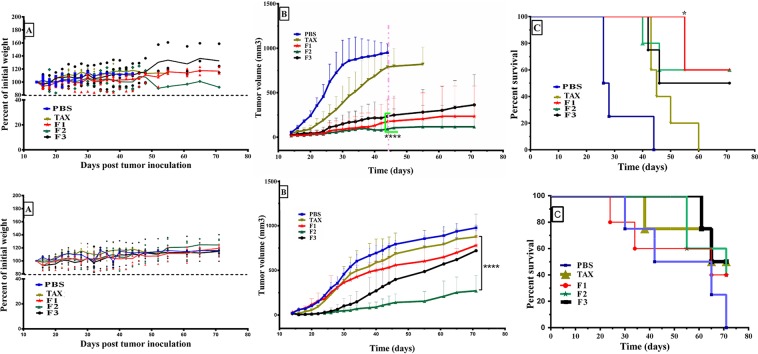
Therapeutic efficacy of DTX loaded liposomes in comparison with control groups (PBS, and Taxotere) in BABL/c models of 4T1 (upper panel) or TUBO (lower panel) mammary carcinoma. A: Body weight, B: Average tumor volume (mm^3^) in all treated groups and C: Survival of all groups was monitored. (n = 4 or 5, mean ± S.D). In 4T1 breast carcinoma tumor model, F1, F2, and F3 formulations significantly extended mouse survival compared to PBS (p < 0.01, p < 0.0, p < 0.05, respectively). Also, the overall survivals were significantly improved in mice treated with F1 liposomes compare to those received Taxotere (p < 0.05, log-rank).

Antitumor efficacy of liposomes containing remotely loaded DTX (8 mg/kg) was evaluated and compared with control groups (Taxotere and PBS) using xenograft models of 4T1 or TUBO mammary carcinoma. For this, body weight, tumor volume (mm^3^), and survival indices of each female BALB/c mouse after treatment were evaluated over the duration of the whole period. The results of weight monitoring indicated no significant loss of mouse body weight over time. Since body weight loss could be used as a sign of toxicity^52^, these results confirm that there is an relatively safe profile in the mice at the test dose for *in vivo* administration (Fig. 4A upper and lower).

Figure 4B (upper) demonstrated that in 4T1 model, liposomal formulations of docetaxel inhibited the rates of tumor growth in terms of mean tumor volume more effectively than Taxotere at the same 8 mg/kg dose despite the PBS group showing the fastest tumor growth. According to our calculation, 38.42 ± 8.8 days after implantation, when the mean tumor volume of the control mice reached to approximately 1000 mm^3^, all of three nanoliposomes exhibited significant inhibition in tumor growth compared to Taxotere (p < 0.0001). Although there weren’t statistically significant difference, F3 treatment showed the most effective antitumor growth effect compared with either F2 or F1, respectively. The survival results demonstrated in a Kaplan–Meier plot (Fig. 4C upper) were used to analyze significant differences in survival rates of different groups. The log-rank survival analysis indicated F1, F2, and F3 extended mouse survival relative to PBS with a statistically significant difference (log-rank test; p < 0.01, p < 0.05, and p < 0.05, respectively). Furthermore, data indicated the comparable values of survival time for mice treated with nanoliposomes and Taxotere. However, in treatment groups, the most improved survival time belonged to mice which received F1 formulation which exhibited significantly more survival time than Taxotere treated group (log-rank test; p < 0.05). Some important indicators relating to the therapeutic efficacy of all groups were presented in Table 4 for tumor models. Mice treated with Taxotere, F1, F2, and F3 formulations at dose of 8 mg/kg showed a median survival time of 43, 71, 71, and 58.5 days, respectively. Liposomal formulations of DTX at dose of 8 mg/kg showed significantly stronger antitumor efficacy than that of Taxotere. In fact, the %TGD of F1, F2, and F3 was 72.83%, 59.38%, 49.69% respectively, whereas the %TGD of Taxotere at the same dose was 25.46. F1, F2, and F3 treatments increased 165.42%, 165.42%, 118.69% the survival time compared to control, which means 44.25 days (F1 and F2) and 31.75 days (F3) increasing in life span in animals receiving a single dose of liposomal formulations 8 mg/kg versus PBS. Therefore, it is clear that liposomal DTX at dose of 8 mg/kg show greater superiority to its corresponding commercial counterpart at equal dose. Based on these data, it could be concluded that although Taxotere offers a clinically relevant concentrated solution and therefore achieves intravenous administration in the commercial DTX formulation, it failed to improve the overall therapeutic efficacy when compared with our liposomes at the same equivalent drug concentration. These results could be attributed to passive cancer targeting which is resulted from an impaired lymphatic network together with leaky tumor blood vessels. These results are consistent with the findings of some other studies, which showed the accumulation of long-circulating nanparticles, at a higher concentration in the tumors than in the plasma or in other tissues^53,54^.

**Table 4:** Therapeutic efficacy data of DTX loaded liposomes and control groups of mice bearing 4T1 or TUBO tumor.

Tumor models	Treatment groups	MST^a^ (day)	TTE^b^ (days ± SD)	TGD^c^ (%)	ILS^d^ (%)
4T1	PBS	26.75	38.42 ± 8.8	0.00	0.00
	Taxotere	43.00	48.20 ± 7.19	25.46	60.75
	F1	71.00	66.40 ± 7.06	72.83	165.42
	F2	71.00	61.23 ± 14.16	59.38	165.42
	F3	58.50	57.50 ± 15.67	49.67	118.69
TUBO	PBS	53.00	54.93 ± 19	0.00	0.00
	Taxotere	68.00	62.75 ± 16	14.24	28.3
	F1	65.00	62.40 ± 16	13.6	22.64
	F2	71.00	68.5 ± 5	24.71	33.96
	F3	68.00	64.6 ± 27	17.61	28.3

^a^Median survival time, ^b^Time to reach end point, ^c^Tumor growth delay, ^d^Increase life span.

In treatment groups of TUBO breast carcinoma model, mice received 8 mg/kg of F2 showed significantly the highest tumor growth inhibition compared to the other groups F1, F3, TAX, and PBS (p < 0.0001). However, tumor inhibition of F1 and F3 formulations was comparable to that of TAX at the same dose and there were no significant differences between them (p > 0.05) (Fig. 4B, lower). Furthermore, MST, TTE, %TGD and ILS of each liposome group was found to be comparable or higher compared to TAX (Table 4). However, the survival results, designated in a Kaplan–Meier plot, showed that there is no significant difference between treatments with nanoliposomes and TAX (p > 0.05) (Fig. 4C, lower).

### Biodistribution study

#### Tumor localization and uptake of iodinated docetaxel loaded liposomes in comparison with free iodinated docetaxel formulated in ethanol

**Figure 5:**
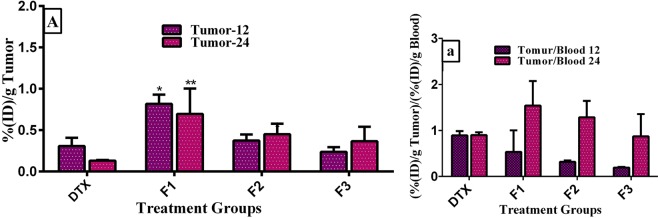
Biodistribution of prepared nanoliposomes and free iodinated docetaxel formulated in ethanol in (A) tumor of BALB/c mice bearing 4T1 tumor at 12 and 24 h post injection of a single dose of 8 mg/kg docetaxel. Panel (a) shows a ratio of radioiodinated docetaxel amount in tumor to blood at each time point. Data are expressed as mean percentage of injected dose per tissue ((%ID)/g) ± SD (n = 3). Statistically significant differences are shown as follows: *p < 0.05, **p < 0.01, ***p < 0.001, ****p < 0.000.1.

**Figure 6:**
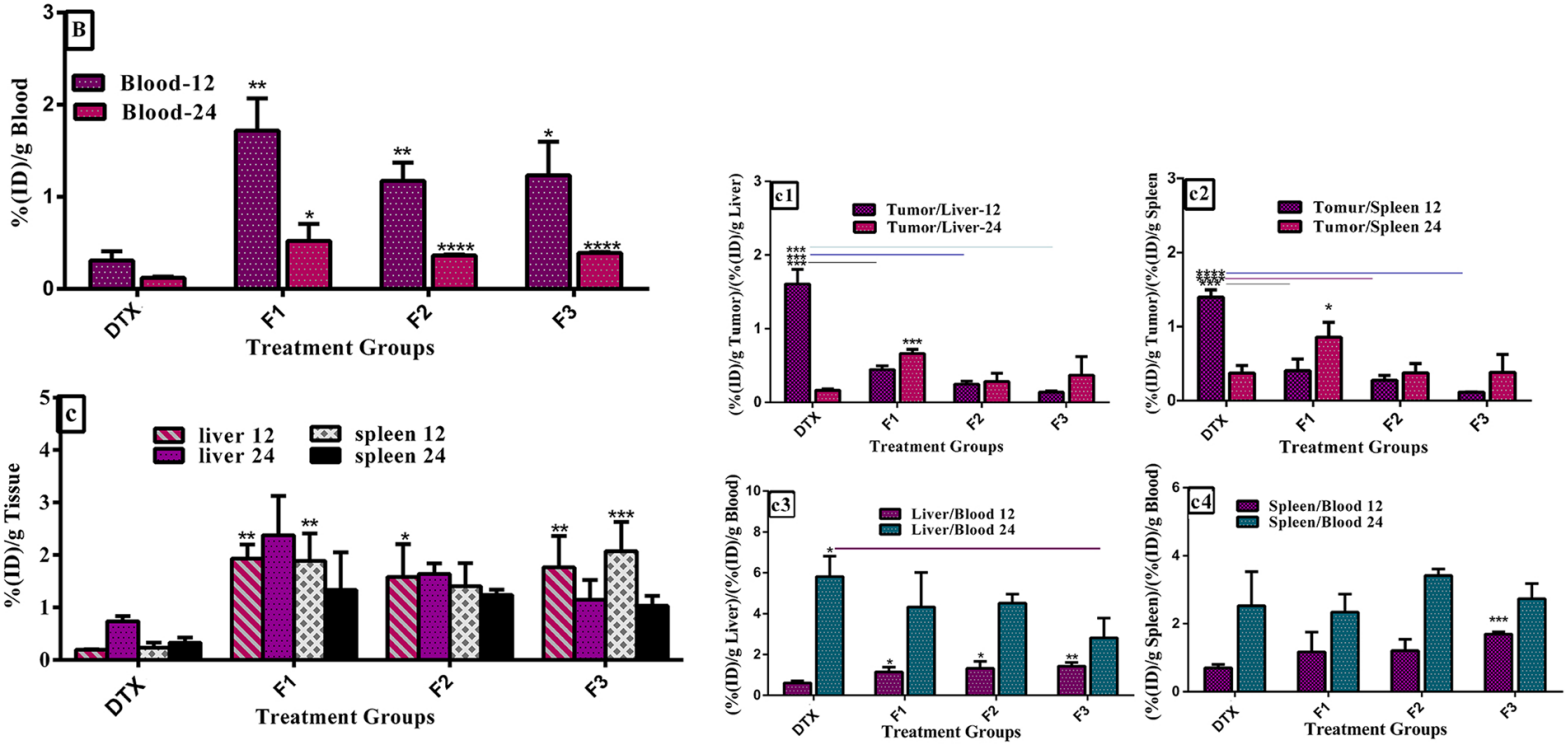
Biodistribution of prepared nanoliposomes and free iodinated docetaxel formulated in ethanol in (B) blood, and (C) liver and spleen of BALB/c mice bearing 4T1 tumor at 12 and 24 h post injection of a single dose of 8 mg/kg docetaxel. Panels (c1) and (c2) show a ratio of radioiodinated docetaxel DTX amount in tumor to liver and spleen (T/NT ratio) at each time point, respectively. Panels (c3) and (c4) show a ratio of radioiodinated docetaxel amount in liver and spleen to blood at each time point, respectively. Data are expressed as mean percentage of injected dose per tissue ((%ID)/g) ± SD (n = 3). Statistically significant differences are shown as follows: *p < 0.05, **p < 0.01, ***p < 0.001, ****p < 0.000.1.

To provide a detailed analysis, biodistribution data of the liposomes and DTX solution was presented separately in tumor, spleen and liver, blood, and other organs. Moreover, we used the analyses including organ to blood ratio and tumor to normal tissue ratio to determine the affinity of formulations to tumoral and non-tumoral tissues (Figs. 5a, 6c1–c4 and 7d1–d8). As shown in Fig. 5, the liposomes displayed higher tumor uptake than the DTX solution. Docetaxel concentration in mice treated with F1 formulation was significantly greater than that of DTX solution after 12 and 24 h post treating (p ≤ 0.05 and p ≤ 0.01 respectively). With F1 formulation, docetaxel accumulated 2.66 and 5.33 folds more in 4T1 tumors than DTX solution after 12 and 24 h post injection respectively. However, the formulations with relatively greater negative surface charges (F2 and F3) accumulated less in tumor than the F1 liposomes. This result is in line with the findings of the survival study which has indicated tumor targeting with F1 formulations significantly could extend mouse survival. Another point that should be noted is increasing trend of the liposomes uptake in tumor tissue compared to DTX solution which exhibited downward trend during this time period. This result could be reasonably attributed to tumor accumulation of nanoliposomes due to the EPR effect and greater decomposition and excretion of DTX solution. Although tumor uptake of the liposomes was greater than that of the DTX solution, the tumor to blood ratio of the solution was greater than that of the liposomes at 12 post injection (Fig. 5a). This inconsistency can be described by the higher blood level of the liposomes than DTX solution.

**Figure 7:**
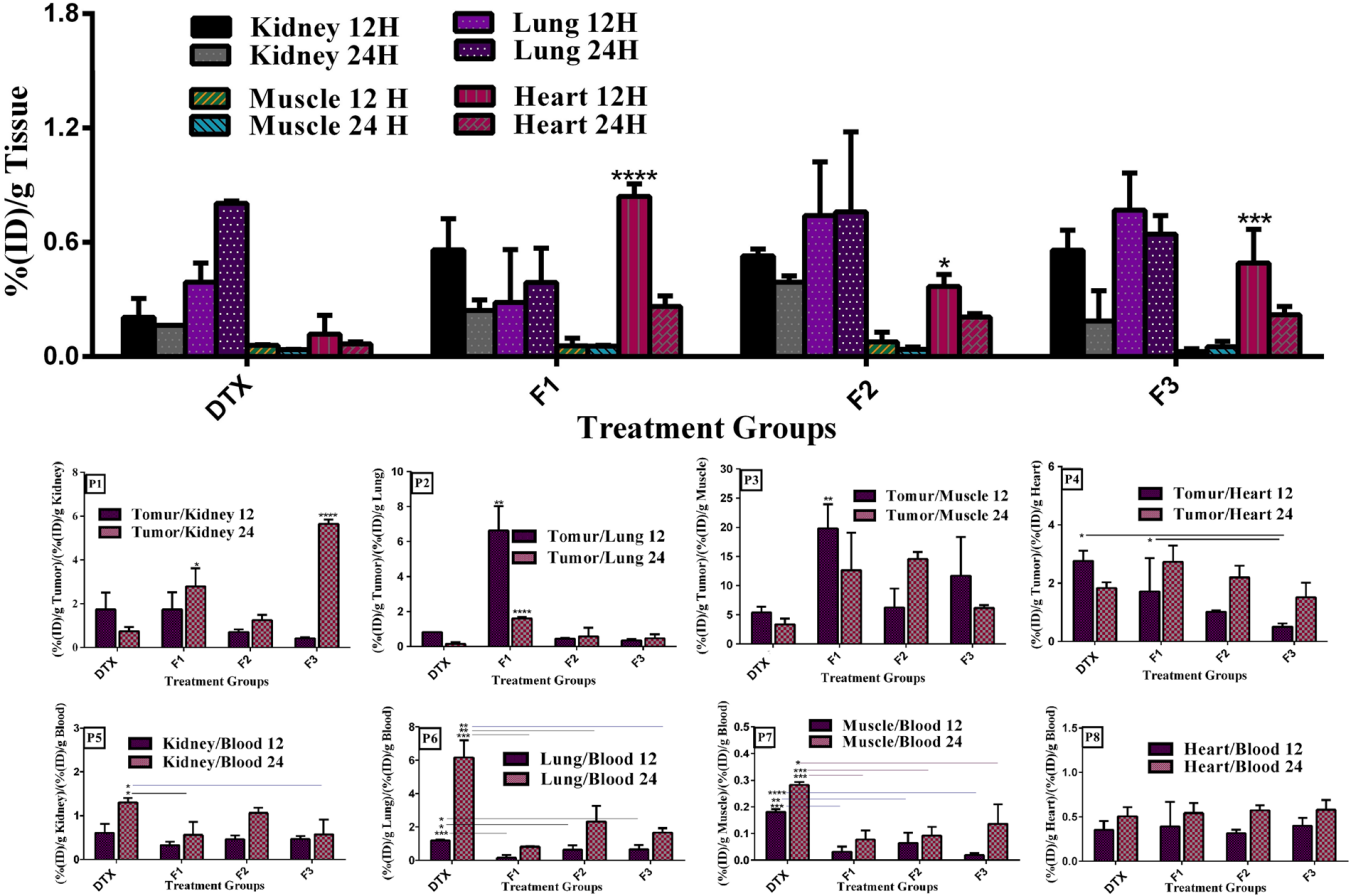
Biodistribution of prepared nanoliposomes and free iodinated docetaxel formulated in ethanol in (D) different organs in BALB/c mice bearing 4T1 tumor at 12 and 24 h post injection of a single dose of 8 mg/kg docetaxel. (D). Panels (d1), (d2), (d3) and (d4) represent a ratio of radioiodinated docetaxel amount in tumor to radioiodinated docetaxel concentration in each organ (T/NT ratio) of each mouse at each time point. Panels (d5), (d6), (d7) and (d8) show a ratio of radioiodinated docetaxel amount in organs to radioiodinated docetaxel concentration in blood of each mouse at each time point. Data are expressed as mean percentage of injected dose per tissue ((%ID)/g) ± SD (n = 3). Statistically significant differences are shown as follows: *p < 0.05, **p < 0.01, ***p < 0.001, ****p < 0.000.1.

#### Biodistribution of iodinated docetaxel loaded liposomes and iodinated docetaxel solution in non-tumoral tissues

The concentrations of docetaxel in blood at 12 and 24 post injections with DTX solution and the NL formulations were shown in Fig. 6B. The blood concentrations of DTX after 12 and 24 h injections show that all three nanoliposomes groups have significantly longer circulation time in blood than DTX solution as a result of the size and stealth property of liposomes. Although there were no significant differences in DTX concentration between liposomal groups, the highest blood concentration of DTX of 1.71 ± 0.35%ID/g, 0.52 ± 0.18%ID/g was obtained with F1 liposomes 12 and 24 h after administration, respectively. Since the reticuloendothelial system (RES) takes up nanoparticles from the blood with higher surface charges more efficiently than those with lower surface charges, it could be attributed to lower surface charge of F1 formulations (ζ –potential: −10.8) compared to that of F2 and F3 formulations (ζ –potential: −12.1, ζ –potential: −13.8, respectively). It should be described that the applying liposomal systems could result in to long circulation time, due to polyethylene glycol (PEG) coating. It is beneficial to tumor targeting *in vivo* and could evidently affect clearance rate of liposomes^55–57^. Also, the spleen and liver of mice treated with nanoliposomes accumulated docetaxel higher than the mice administrated with DTX solution (Fig. 6C). At 12 and 24 h after injection, the amount of DTX in liver for F1, F2, and F3 was 10.03 and 3.22 folds, 8.21 and 2.22 folds, and 9.16 and 1.55 folds higher than the DTX solution, respectively. At these time intervals, DTX concentrations in spleens of F1, F2, and F3 injected mice were 6.47 and 5.74 folds, 4.33 and 5.30 folds, and 6.41 and 4.42 folds higher than those treated with the DTX solution, respectively. These significant differences have been nonsignificant over the time which could explain the higher rate of DTX (in the solution) clearance from blood and its uptake by RES (Fig. 6B,C). In contrary to the accumulation results in tissues rich in cells of the RES, the uptake of DTX formulated with ethanol in kidneys, lungs, and muscle was comparable with that of nanoliposomes at either 12 or 24 h after treatment (Fig. 7). However, docetaxel concentration of F2 and F3 formulations in heart was higher than that of DTX solution at 12 and 24 h after i.v. tretment. These levels of increase were statistically significant for F2 at 12 h (p < 0.05) and for F3 at 12 h and 24 h (p < 0.05 and p < 0.01 respectively). These results are in line to their trends presented in biodistribution results of DTX solution and the NL formulations in blood. It could be attributed to longer blood circulation behavior of F2 and F3 over F1 and the DTX solution which may practically cause the nonspecific extravasations in non-tumoral organs such as heart (Fig. 7D). The most important findings in the distribution analysis of normal tissues were comparable concentration of docetaxel in organs of mice received the formulations and mice treated with DTX solution, and longer blood circulation time of nanoliposomes compared to the solution.

Tumor to normal tissue ratio analyses also demonstrated higher inclination of DTX dissolved in ethanol to different organs compared to liposomal formulations (Fig. 7d5–d8). According to the results of this study, our formulations had overall higher trends to tumor compared to DTX solution. It could be concluded that in mice treated with our nanoliposomal formulations, the tumoral concentration of docetaxel was greater than all normal organs with the exception of the liver, spleen, and lung (for F2 and F3) after 24 h.

It may be thought that the biodistribution test should be performed ideally with a method which can evaluate the localization and uptake of the nanoliposomes versus that of the current clinical dosage form of DTX, i.e. Taxotere.

## Conclusion

In this study, DTX was encapsulated in nanoliposomes based on a new remote loading method using mannitol and acetic acid as hydration buffer for the chemotherapy of breast carcinoma. Physicochemical analysis of DTX-liposomes revealed that liposomes have appropriate size and zeta potential to target the tumor with EPR mechanism. HPLC analysis revealed that the DTX-liposomes had high encapsulation efficiency and DTX concentration and indicating that remote loading based on mannitol and acetic acid could be reported as a promising strategy of DTX encapsulation. Passive loaded counterparents liposomes showed three times lower encapsulation efficacy compared to the remote loaded liposomes. Moreover, the DTX-liposomes were remarkably stable in plasma and physiologic pH, suggesting that the liposomal formulations are appropriate for delivery of encapsulated DTX to tumors without significant leakage into the blood. The biodistribution of iodinated DTX as free or liposomal form exhibited significantly greater accumulation of DTX-liposomes in tumors than that of free docetaxel due to the EPR effect. Between nanoliposomal formulations, F1 displayed significantly more abundant DTX in tumors (p < 0.05). DTX-nanoliposomes were also able to provide prolonged circulation. *In vivo* experiment with BALB/c mice bearing 4T1 or TUBO breast carcinoma tumors also showed that DTX-liposomes could significantly delay tumor growth and prolonged the survival time in comparison with control and Taxotere groups. The animal data fully demonstrated the rationale of utilization of rigid, pegylated liposomes and the potential of these liposomes in the treatment of breast cancer. Therefore, our results could confirm high efficiency of DTX delivering and a very low level of drug losing by the liposomes compared to commercially available product, Taxotere. Among the formulations, F1 and F2 formulations were stable and showed good anti-tumor activity and merits further investigation.

## Material and Methods

### Materials

Taxotere vial was obtained from Sanofi Aventis Company (France). 1,2-distearoylsn- glycero-3 phosphoethanolamine-N-[methoxy (polyethylene glycol)- 2000] (mPEG2000-DSPE), hydrogenated soy phosphatidylcholine (HSPC), 1,2-distearoyl-sn-glycero-3-[Phospho-rac-(1-glycerol)] (Sodium Salt) (DSPG), 1,2-dipalmitoyl-sn-glycero-3-[Phospho-rac-(1-glycerol)] (Sodium Salt) (DPPG) were from Lipoid (Ludwigshafen, Germany). Cholesterol (Chol) was purchased from Sigma-Aldrich (Steinheim, Germany). [3-(4, 5-dimethylthiazol-2-yl)-2], diphenyltetrazolium bromide (MTT) was from Promega (Madison, WI). Iodine 125 (^125^I) was purchased from Amersham (Uppsala, Sweden). MCF-7, 4T1, TUBO breast cancer cells and NIH/3T3 fibroblast normal cells were provided by American Type Culture Collection (ATCC). All solvents and reagents used in this study were HPLC grade.

### Preparation of docetaxel loaded liposomes

Liposome formulations were prepared by thin film hydration and extrusion methods based on passive loading and remote loading strategies (Supplementary Table S2B). To prepare the remote loaded liposomes, the lipids as presented in Table 1 were dissolved in chloroform (total lipid concentration: 100 mM). Then, the lipid solution in chloroform was dried under a rotary evaporator and then followed by a high vacuum overnight. For the preparation of 1 ml of remote loaded liposomes, the lipid films were hydrated with 1 ml of hydration buffer solutions listed in Supplementary Table S2B at 60 °C and then, extruded through polycarbonate membranes with diameter of 200, 100 and 50 nm (11 times for each filter). Afterward, the solutions were transferred to a dialysis tube (cut off 12–14 kDa) and dialyzed for 2 days against dextrose 5% (pH 7.00) at room temperature to remove the hydration buffer from liposomes. Finally, the liposomes were loaded by docetaxel, to prepare docetaxel loaded liposomes. For this purpose, ethanol solution containing docetaxel (10 mg/mL) was added to liposome composition at 67 °C (at lipid-to-drug molar ratio of 25:1). After 120 minutes incubation at 67 °C, the liposomes were cooled down to the room temperature. The mixtures were incubated overnight at 4 °C and again were dialyzed for 2 days against the dextrose 5% (pH 7.00) at room temperature to separate free DTX from the liposomes. Finally, the formulations were filtered through 0.45 µm syringe filter to sterilize the formulations and separate the possible precipitates of DTX.

For passive loading methods, phospholipids and cholesterol in chloroform, and DTX dissolved in ethanol were added in round-bottom flasks. The mixture was dried using a rotary evaporator, and then a freeze-dryer. The lipid films were hydrated with mannitol and acetic acid buffer solution, followed by overnight incubation at 4 °C. Then, liposomes were extruded through polycarbonate filters (200 to 50 nm pore size) using extruder device. For the purification of DTX loaded liposomes from free ones, the resulting liposomes were dialyzed using 12–14 kDa molecular weight cut off dialysis membrane against dextrose 5% four times for 8 h each, at room temperature. The final liposomal formulations were filtered through a 0.45 μm syringe filter as mentioned above.

### Formulation development

The first step in liposome preparation was optimizing of variables including incubation time, cholesterol concentration, and incubation temperature to choose ideal parameters for higher encapsulation efficacy. As shown in Supplementary Table S2A, this part of our experiment was performed with lipid compositions of HSPC/mPEG 2000-DSPE/DSPG/Chol (F3). Based on the characterization of these formulations, incubation temperature of 67 °C, cholesterol molar ratio of 20%, and incubation time of 2 hours provided the highest amount of encapsulation efficacy (~100). However, there was a crystallization behavior in suspension after some days when the cholesterol molar ratios were 20 and 15%. Therefore, we selected 10% molar ratio for cholesterol concentration in which the formulations had a desired encapsulation efficacy with acceptable stability. Afterward, we have prepared twelve different formulations based on passive or active loading strategies. As shown in Supplementary Table S2B, the formulations were hydrated with the various buffer solutions at incubation temperature of 67 °C. Our results showed that formulations of HSPC/mPEG2000-DSPE/Chol (F1) and HSPC/mPEG 2000-DSPE/DPPG/Chol (F2) have also a good encapsulation of DTX at molar ratio of 10% for cholesterol. Therefore, 10% molar ratio of cholesterol, incubation temperature of above 66 °C (67 °C for this work), and incubation time of 2 hours were chosen as the most optimal parameters (Supplementary Table S2A). It is worthy to note that incubation temperatures and incubation times below 66 °C and 2 hours, respectively, were also considered for drug loading evaluation that resulted in low amount of encapsulation efficacy.

### Characterization of docetaxel loaded liposomes

#### Size, zeta potential, DTX content, and drug encapsulation efficiency

The mean particle size (Z-average (nm)), polydispersity index (PDI), and ζ potential (mV) were measured by the dynamic light scattering (ZetaSizer Nano-ZS; Malvern Instruments Ltd., United Kingdom). Samples were prepared by adding 990 µl dextrose 5% of pH 7.00 to 10 µl nanoliposomes suspension until the counter rate was less than 500 Kcps (Kilo counts per second). The data were obtained with measurements performed at least in triplicates.

The morphological characteristics of liposomes were imaged by a Leo 912 AB transmission electron microscope (Zeiss, Jena, Germany) at a voltage of 120 Kv. To prepare samples for TEM, 20 microliter of diluted liposomes samples (1:100 of sample to PBS buffer) was placed on a carbon-coated copper grid and then the negatively staining was done on the formed thin film of samples on the grid by adding 2% filtered uranyl acetate (w/v) (pH 7.00). The resultant stained samples were finally air-dried and imaged^58^.

The drug content in the DTX loaded NLs was measured by the high performance liquid chromatography (HPLC). Knauer smart line HPLC (Berlin, Germany) was equipped with a Waters C18, 3.5 μm, 150 × 4.6 mm, 100 A° column (Knauer) and an UV detector (Knauer S2600) set at 230 nm. Briefly, DTX loaded NLs were dissolved in ethanol and incubated 20 minutes at 66 °C and then extracted by mobile phase consisting of deionized water and acetonitrile according to Supplementary Table S3. The solution was transferred into HPLC vial after filtering through 0.22 mm syringe filter. The flow rate of mobile phase was 1 ml/min and the run time was 45 min for each sample. The column effluent was detected at 230 nm with an UV/VIS detector (Supplementary Fig S1). The calibration curve was linear in the range of 125–5,000 µg/ml (Supplementary Table S4) with a correlation coefficient of R^2^ 0.9999 (Supplementary Fig. S2). The amount of DTX entrapped in liposomes was measured by HPLC and compared with the reference standard curve using serial dilution of DTX. The entrapment efficiency (EE) was calculated according to the following equations.

$${\rm{EE}}( \% )=\frac{{\rm{amount}}\,{\rm{of}}\,{\rm{DTX}}\,{\rm{entrapped}}\,{\rm{in}}\,{\rm{liposomes}}}{{\rm{amount}}\,{\rm{of}}\,{\rm{total}}\,{\rm{DTX}}\,{\rm{applied}}\,{\rm{in}}\,{\rm{the}}\,{\rm{preparation}}}\times 100 \% $$

#### *In vitro* drug release

Release test in our study was performed according to two protocols. The first drug release experiment was performed in 50% fresh human plasma. For this, after plasma decomplementing by heating at 60 ^o^C for 10 min, 5% dextrose was added to the plasma (1:1 v/v) to prepare 50% plasma as release media. The drug loaded liposomes and their control counterparts (liposomes without drug) were added to this media (1: 9 v/v) and incubated at 37 ^o^C. At 0, 0.5, 1, 2, 4, 6, 12, 24, 48, and 72 hours intervals, 1 ml release medium were removed from the released media. The samples were transferred to an Amicon-Ultra centrifuge filter device (Millipore Billerica, MA) with MWCO of 100 kD and centrifuged at 9000 × g for 10 min. Filtrates were collected and diluted by ethanol (1:2 v/v). After centrifugation at 14000 × g for 1.5 min, the supernatants were assayed for released DTX. The error bars were obtained from duplicate samples.

The second release study was conducted using a dialysis method in three different media with the pHs of 5.5, 6.5, and 7.00 to simulate the endosomal, tumoral and plasma condition, respectively. Briefly, DTX loaded NLs (100 ul) were put into a Cellulose Dialysis Membrane (cut off 12–14 kDa) and then, the closed bag immersed in 100 ml of phosphate - buffered dextrose 5% as a release medium and incubated at 37 °C. At 0, 1, 2, 4, 6, 12, 24, 48, and 72 hours intervals, 1 ml release medium were removed from the released media and replaced with the same volume of fresh medium. For evaluation of the drug content in the samples, 20 ul of samples were injected into the column and measured by the same HPLC procedure as mentioned above. Finally, the results were multiplied by dilution factor to achieve the amount of DTX released from entrapped liposomes in the closed bags. The error bars were obtained from triplicate samples.

#### Stability tests

Stability test is a very meaningful parameter for storage and transportation of a colloidal system. Size, PDI, zeta (ζ) potential, and leakage stability of liposomes maintained at 4 °C were measured at 0, 1, 3, 6 months intervals by the same DLS, and HPLC procedures as mentioned above.

### *In vitro* cytotoxicity of NLs

TUBO, a cloned cell line overexpressing the rat HER2/neu protein, was grown in 25 cm^2^ cell culture flask maintained at 37 °C in a humidified environment of 5% CO2. Dulbecco Modified Eagle Medium (DMEM) medium supplemented with 20% (v/v) Fatal Calf Serum (FCS), 100 U/ml penicillin and 100 mg/ml streptomycin was replenished every two days. After 90% confluence, the cells were collected by trypsinization using Trypsin solution and cultured in 96-well plate at a density of 5000 cell/well. After overnight incubation which the cells reached confluence, the nanoliposomes and Taxotere were dispersed in the medium at concentration of 500, 250, 120, 62.50, 31.25, and 15.62 µg/ml. The wells with nanoliposomes and Taxotere were incubated at 37 °C for 24, 48, and 72 h. Moreover, culture of NIH/3T3 cells in DMEM medium supplemented with 10% FCS, human breast adenocarcinoma cell line MCF-7 cells and 4T1 cells in RPMI-1640 medium supplemented with 10% FCS, and their treatments with formulations and Taxotere were performed in the same way. The viability of cells was determined using a MTT test. Then, IC_50_ of formulations was calculated by CalcuSyn version 2.0 (CalcuSyn Software, USA). IC_50_ is the drug concentration needed for 50% cell viability in a designated time period of the cell culture^59^.

### Animal studies

Four to six week-old female BALB/c mice were supplied from the Pasteur Institute (Tehran, Iran) for human breast adenocarcinoma model. The animal experiments were carried out according to the rules established by the National Insinuate for Medical Research Development (IR.NIMAD.Rec.1396.360), and approved by the ethics committee of Tehran University of Medical Sciences under the protocol number of 963392. The animals were acclimatized at temperature of 25 ± 2 °C in a colony room with free access to water and animal food.

### Chemotherapy study

On day 0, 3 × 10^5^ 4T1 or TUBO cells suspended in 60 μl PBS were subcutaneously administered in the right flank of each BALB/c mouse. 10 days after implantation when the tumor volume was about 6 mm^3^ more or less, mice were randomly distributed into five different groups and each group received its specific treatment. Treatment groups (n = 4 or 5/group) included PBS, Taxotere, and three different nanoliposomal formulations. The mice were intravenously treated with DTX at a dose of 8 mg/kg of body weight via tail vein.

Tumor volume was estimated by measuring the tumor in three orthogonal diameters (a, b, and c) according to the formula: tumor volume = (length × width × height) × 0.52 mm^3^. The mice were sacrificed if the size of tumor was >1000 mm^3^ or body weight loss >20% of their initial wight^60^. The time to reach end point (TTE) for each mouse was calculated based on the line equation obtained by exponential regression of the tumor growth curve. The percent of tumor growth delay (%TGD) was obtained from following formula: %TGD = (the mean TTE of treatment group - the mean TTE of the control group)/the mean TTE of the control group ×100^60,61^. Furthermore, increased life span (%ILS) for each treatment group was calculated according to the following equation: %ILS = (mean survival time of treatment group/mean survival time of PBS group × 100)−100^62,63^.

### Biodistribution test

#### Radioionization of DTX

Docetaxel was labeled with radioactive iodine (^125^I) according to the modified method of Johnstone and Thorpe^64^. Briefly, 200 mg of DTX in ethanol (247 uM) was added in a round bottom flask, then 2 μL Na^125^I (200 μCi) solution as an oxidizing agent 80 μL Chloramine T were added. The reaction mixture was stirred for 30 seconds with a magnetic stirrer and incubated in a 65 ^o^C bath water for 30 min. The reaction was stopped by adding 1 mg of sodium metabisulfite. The radioiodinated docetaxel were purified using Sephadex G-50 (Sigma– Aldrich) column chromatography to separate the iodine from iodinated DTX. The drug was labeled with specific activities of 10 mCi/mg and then used for the preparation of liposomes.

#### Biodistribution study

10 days after implantation when the tumor volume was about approximately 5 mm^3^ more or less, Balb/c mice (3 per group) were injected with 1 μCi for time point of 12 h and 2 μCi for time point of 24 h. ^125^I-labeled DTX loaded liposomal formulations and purified solution of ^125^I-labeled DTX were administrated via the tail vein and the mice of each group were euthanized in 12 h or 24 h after injection for tissue collection. Organs were removed, washed with PBS buffer, weighted, and the radioactivity of each organ was measured by means of a gamma counter (Delshid, Tehran, Iran). The results were presented as mean percentage of injected dose per gram of tissue (%ID/g).

### Statistical analysis

GraphPad Prism version 5 (GraphPad Software, San Diego, CA) was used to analyses the data. The average of data was shown as the mean ± SD (standard deviation). Survival data were analyzed by the log-rank (Mantel–Cox) test. To check for the statistically significant differences between groups, two-way ANOVA and Tukey’s post-test were employed. A p-value of <0.05 was considered significant.

## Supplementary information

Supplementary Figure S1.

Supplementary Figure S2.

Supplementary Table S1.

Supplementary Table S2A.

Supplementary Table S2B.

Supplementary Table S3.

Supplementary Table S4.
