# Supplementary material for: Optimization of Docetaxel Loading Conditions in Liposomes: proposing potential products for metastatic breast carcinoma chemotherapy
Source: Sci Rep. 2020 Mar 27;10:5569. doi: 10.1038/s41598-020-62501-1 (PMC7101339; doi:10.1038/s41598-020-62501-1)
Supplement: Supplementary file 4 — Supplementary Table S2A. [file 41598_2020_62501_MOESM4_ESM.pdf]

Optimization of Docetaxel Loading Conditions in Liposomes: proposing potential products for metastatic breast carcinoma chemotherapy

Roghayyeh Vakili-Ghartavol[1], Seyed Mahdi Rezayat [1, 2], Reza Faridi-Majidi[1], Kayvan Sadri[3], Mahmoud Reza Jaafari*[4, 5]

*Corresponding author: Mahmoud Reza Jaafari; Email: Jafarimr@mums.ac.ir

[1]Department of Medical Nanotechnology, School of Advanced Technologies in Medicine, Tehran University of Medical Sciences, Tehran, Iran.

[2] Department of Pharmacology, School of Medicine, Tehran University of Medical Sciences, Tehran, Iran.

[3]Nuclear Medicine Research Center, Mashhad University of Medical Sciences, Mashhad 98451-3546, Iran

[4]Nanotechnology Research Center, Pharmaceutical Technology Institute, Mashhad University of Medical Sciences, Mashhad, Iran

[5]Department of Pharmaceutical Nanotechnology, School of Pharmacy, Mashhad University of Medical Sciences, Mashhad, Iran

Supplementary Table S2A

Optimization of important parameters involved in encapsulation efficacy of DTX into the HSPC/ DSPG/ DSPE-mPEG 2000/ Chol formulation.

Incubation Time	Molar Ratio of Cholesterol (%)	Incubation Temperature (°C)	Encapsulation Efficacy (%)
2 (h)	50	67	0
2 (h)	45	67	5
2 (h)	40	67	10
2 (h)	35	67	30
2 (h)	30	67	40
2 (h)	20	67	100
2 (h)	15	67	86
2 (h)	10	67	67
5 (min)	20	67	2
10 (min)	20	67	4
20 (min)	20	67	10
30 (min)	20	67	10
40 (min)	20	67	10
50 (min)	20	67	30
1 (h)	20	67	40
1.5 (h)	20	67	60
2 (h)	20	67	100
2 (h)	20	50	2
2 (h)	20	55	5
2 (h)	20	57	5
2 (h)	20	60	10
2 (h)	20	63	15
2 (h)	20	65	17
2 (h)	20	66	30
2 (h)	20	67	100
2 (h)	20	68	100
