# Supplementary material for: Optimization of Docetaxel Loading Conditions in Liposomes: proposing potential products for metastatic breast carcinoma chemotherapy
Source: Sci Rep. 2020 Mar 27;10:5569. doi: 10.1038/s41598-020-62501-1 (PMC7101339; doi:10.1038/s41598-020-62501-1)
Supplement: Supplementary file 5 — Supplementary Table S2B. [file 41598_2020_62501_MOESM5_ESM.pdf]

Optimization of Docetaxel Loading Conditions in Liposomes: proposing potential products for metastatic breast carcinoma chemotherapy

Roghayyeh Vakili-Ghartavol[1], Seyed Mahdi Rezayat [1, 2], Reza Faridi-Majidi[1], Kayvan Sadri[3], Mahmoud Reza Jaafari*[4, 5]

*Corresponding author: Mahmoud Reza Jaafari; Email: Jafarimr@mums.ac.ir

[1]Department of Medical Nanotechnology, School of Advanced Technologies in Medicine, Tehran University of Medical Sciences, Tehran, Iran.

[2] Department of Pharmacology, School of Medicine, Tehran University of Medical Sciences, Tehran, Iran.

[3]Nuclear Medicine Research Center, Mashhad University of Medical Sciences, Mashhad 98451-3546, Iran

[4]Nanotechnology Research Center, Pharmaceutical Technology Institute, Mashhad University of Medical Sciences, Mashhad, Iran

[5]Department of Pharmaceutical Nanotechnology, School of Pharmacy, Mashhad University of Medical Sciences, Mashhad, Iran

Supplementary Table S2B

Characteristics of DTX loaded liposomal formulations prepared based on different methods.

No	Preparation Method	Formulations -Hydration Buffer	Z Average [c] (nm)	PDI[d]	Zeta potential (mV)	EE[e] (%)
**1**	Passive Loading	HSPC/mPEG 2000-DSPE/ Chol, (8.5/0.5/1) -Mannitol 290 mM, Acetic acid 10mM	125	0.087	-10.3	20
**2**	Passive Loading	HSPC/mPEG2000-DSPE/DPPG/Chol (85/5/5/10) -Mannitol 290 mM, Acetic acid 10mM	139.0	0.156	-10.9	18
**3**	Passive Loading	HSPC/mPEG2000-DSPE/DSPG/Chol (85/5/5/10) -Mannitol 290 mM, Acetic acid 10mM	108.4	0.178	-13.7	26
**4**	Remote Loading	HSPC/ mPEG 2000-DSPE/Chol, (8.5/0.5/1) -Sucrose 10%, HEPES 10 mM	110	0.121	-9.32	23
**5**	Remote Loading	HSPC/ mPEG 2000-DSPE/ Chol, (8.5/0.5/1) -mannitol 290 mM, succinate sodium 10 mM	109.5	0.157	-10.6	16.6
**6**	Remote Loading	HSPC/ mPEG 2000-DSPE/ Chol, (8.5/0.5/1) -mannitol 290 mM, acetic acid 10 mM	113.5	0.152	-8.52	67
**7**	Remote Loading	HSPC/ mPEG 2000-DSPE/ Chol, (7.5/0.5/2) - mannitol 290 mM, acetic acid 10 mM	109.6±8	0.165	-10.8	20
**8**	Remote Loading	HSPC/mPEG2000-DSPE/DSPG:Chol, (8/0.5/0.5/1) - mannitol 290 mM, acetic acid 10 mM	113.1±5	0.145	-12.1	71.8
**9**	Remote Loading	HSPC/mPEG 2000-DSPE/DPPG/Chol, (8/0.5/0.5/1) - mannitol 290 mM, acetic acid 10 mM	118.1±10	0.198	-13.8	40.6
**10**	Remote	HSPC/ mPEG 2000-DSPE/Chol, (6/0.5/3.5)-	-[f]	-	-	10

	Loading	mannitol 290 mM, acetic acid 10 mM				
**11**	Remote Loading	HSPC/mPEG2000-DSPE/DSPG/Chol, (5.5/0.5/0.5/3.5) -mannitol 290 mM, acetic acid 10 mM	-	-	-	10
**12**	Remote Loading	HSPC/mPEG 2000-DSPE/DPPG/Chol, (5.5/0.5/0.5/3.5) -mannitol 290 mM, acetic acid 10 mM	-	-	-	10

[a] Hydration buffer
[b] Dialysis buffer
[c] Values are presented from triplicate measurements of each formulation
[d] Poly dispersity index
[e] Encapsulation efficiency
[f] Values were not measured because of low EE
