# Supplementary material for: Optimization of Docetaxel Loading Conditions in Liposomes: proposing potential products for metastatic breast carcinoma chemotherapy
Source: Sci Rep. 2020 Mar 27;10:5569. doi: 10.1038/s41598-020-62501-1 (PMC7101339; doi:10.1038/s41598-020-62501-1)
Supplement: Supplementary file 6 — Supplementary Table S3. [file 41598_2020_62501_MOESM6_ESM.pdf]

Optimization of Docetaxel Loading Conditions in Liposomes: proposing potential products for metastatic breast carcinoma chemotherapy

Roghayyeh Vakili-Ghartavol[1], Seyed Mahdi Rezayat [1, 2], Reza Faridi-Majidi[1], Kayvan Sadri[3], Mahmoud Reza Jaafari*[4, 5]

*Corresponding author: Mahmoud Reza Jaafari; Email: Jafarimr@mums.ac.ir

[1]Department of Medical Nanotechnology, School of Advanced Technologies in Medicine, Tehran University of Medical Sciences, Tehran, Iran.

[2] Department of Pharmacology, School of Medicine, Tehran University of Medical Sciences, Tehran, Iran.

[3]Nuclear Medicine Research Center, Mashhad University of Medical Sciences, Mashhad 98451-3546, Iran

[4]Nanotechnology Research Center, Pharmaceutical Technology Institute, Mashhad University of Medical Sciences, Mashhad, Iran

[5]Department of Pharmaceutical Nanotechnology, School of Pharmacy, Mashhad University of Medical Sciences, Mashhad, Iran

Supplementary Table S3
HPLC gradient program based on deionized water and acetonitrile for DTX analysis

Time (min)	F.R (ml/min)	Water (%)	Acetonitrile (%)
0	1	72	28
9	1	72	28
39	1	28	72
39.1	1	72	28
45	1	72	28
