# Supplementary material for: Optimization of Docetaxel Loading Conditions in Liposomes: proposing potential products for metastatic breast carcinoma chemotherapy
Source: Sci Rep. 2020 Mar 27;10:5569. doi: 10.1038/s41598-020-62501-1 (PMC7101339; doi:10.1038/s41598-020-62501-1)
Supplement: Supplementary file 7 — Supplementary Table S4. [file 41598_2020_62501_MOESM7_ESM.pdf]

Optimization of Docetaxel Loading Conditions in Liposomes: proposing potential products for metastatic breast carcinoma chemotherapy

Roghayyeh Vakili-Ghartavol[1], Seyed Mahdi Rezayat [1, 2], Reza Faridi-Majidi[1], Kayvan Sadri[3], Mahmoud Reza Jaafari*[4, 5]

*Corresponding author: Mahmoud Reza Jaafari; Email: Jafarimr@mums.ac.ir

[1]Department of Medical Nanotechnology, School of Advanced Technologies in Medicine, Tehran University of Medical Sciences, Tehran, Iran.

[2] Department of Pharmacology, School of Medicine, Tehran University of Medical Sciences, Tehran, Iran.

[3]Nuclear Medicine Research Center, Mashhad University of Medical Sciences, Mashhad 98451-3546, Iran

[4]Nanotechnology Research Center, Pharmaceutical Technology Institute, Mashhad University of Medical Sciences, Mashhad, Iran

[5]Department of Pharmaceutical Nanotechnology, School of Pharmacy, Mashhad University of Medical Sciences, Mashhad, Iran

Supplementary Table S4

DTX concentrations and correspond Area Under the DTX concentration – time Curves (AUC) obtained from HPLC analysis.

Sample number	AUC	DTX concentration
1	63577833.9	15.6
2	113169183	31.2
3	201391383	62.5
4	359055883	125
5	680722583	250
6	2554465681	1000
